# The Use of Contrast-Enhanced Multidetector Computed Tomography Imaging in Advanced Abdominal Pregnancy

**DOI:** 10.7759/cureus.22542

**Published:** 2022-02-23

**Authors:** Adrian Chan, Fidel S Rampersad, Ashton Ramsundar, Anil Boodram, Robbie Rampersad

**Affiliations:** 1 Department of Radiology, The University of the West Indies, St. Augustine, TTO; 2 Department of Radiology, Eric Williams Medical Sciences Complex, Champ Fleurs, TTO; 3 Department of Radiology, Port of Spain General Hospital, Port of Spain, TTO

**Keywords:** embolization, obstetric, advanced abdominal pregnancy, ectopic pregnancy, computed tomography abdomen

## Abstract

An advanced abdominal pregnancy is defined as an extrauterine pregnancy over twenty weeks gestation with a fetus living, or showing evidence of having once lived, in the mother’s abdominopelvic cavity. Our case is a 35-year-old patient with a 23-week extrauterine pregnancy, with a congenital head defect (scaphocephaly and hydrocephalus), located in the left side of the maternal abdomen with a period of gestation of 23 weeks, who underwent preoperative imaging with contrast-enhanced multidetector computed tomography (CE-MDCT). CT imaging provided significant information on the placenta and its arterial supply/venous drainage and confirmed the presence of an arteriovenous malformation of the right uterine artery. CT imaging also allowed planning of preoperative uterine artery coil embolization.

## Introduction

An advanced abdominal pregnancy is regarded as a rare form of ectopic pregnancy with an incidence ranging from one in 400 to one in 50,000 deliveries [1]. It is defined as a pregnancy over 20 weeks gestation with fetus living, or showing evidence of having once lived and developed, in the mother’s abdominal cavity [1]. Although ultrasound is regarded as the main imaging in the diagnosis of abdominal pregnancy, cross-sectional imaging such as CT can assist in the diagnosis and preoperative assessment as well as intraoperative planning, which can significantly decrease patient morbidity and mortality [2,3]. Within the literature, placental management is essential as significant hemorrhage can occur during surgery with preoperative uterine artery embolization and methotrexate therapy being an alternate viable option [4]. Our case report highlights the importance of contrast-enhanced multidetector computed tomography (CE-MDCT) and preoperative uterine embolization in the management of advanced abdominal pregnancy and their role in preoperative planning.

## Case presentation

This a 35-year-old gravida 3, para 2 with an unremarkable past obstetric history, presented to the antenatal clinic in mid-pregnancy with no prior antenatal visits. The patient then presented three weeks later to the emergency department with lower abdominal pain. Obstetric ultrasound was then done which showed an extrauterine pregnancy located in the left pelvis, with cardiac activity and fetal scaphocephaly and hydrocephalus, with an estimated period of gestation of 23 weeks. There was no free intra-abdominal fluid. The patient was counseled and then underwent further cross-sectional imaging with contrast-enhanced multidetector computed tomography (CE-MDCT) for preoperative assessment.

The imaging examination was performed on a multislice (16-detector) row CT scanner, scanning protocol: beam collimation 16x0.625 mm, and section thickness of 1.25 mm. The area of scan coverage ranged from the lower chest to the proximal femur. A non-contrast thick slice acquisition was performed prior to the contrast injection. One hundred and twenty milliliters (120 mL) of a low osmolar contrast medium (iopromide 300 mg I/mL) followed by 50 mL of saline, which was injected via a double power injector into the patient's left antecubital vein. A bolus tracking technique of the thoracic abdominal aorta threshold of 100 HU over baseline was used to ensure maximum arterial enhancement. A scan delay acquisition was performed to demonstrate the venous supply. In addition to 2D axial views, a number of 3D reconstructions were generated. These reconstructions included multiplanar reformation (MPR) and maximum intensity projection (MIP).

**Figure 1:**
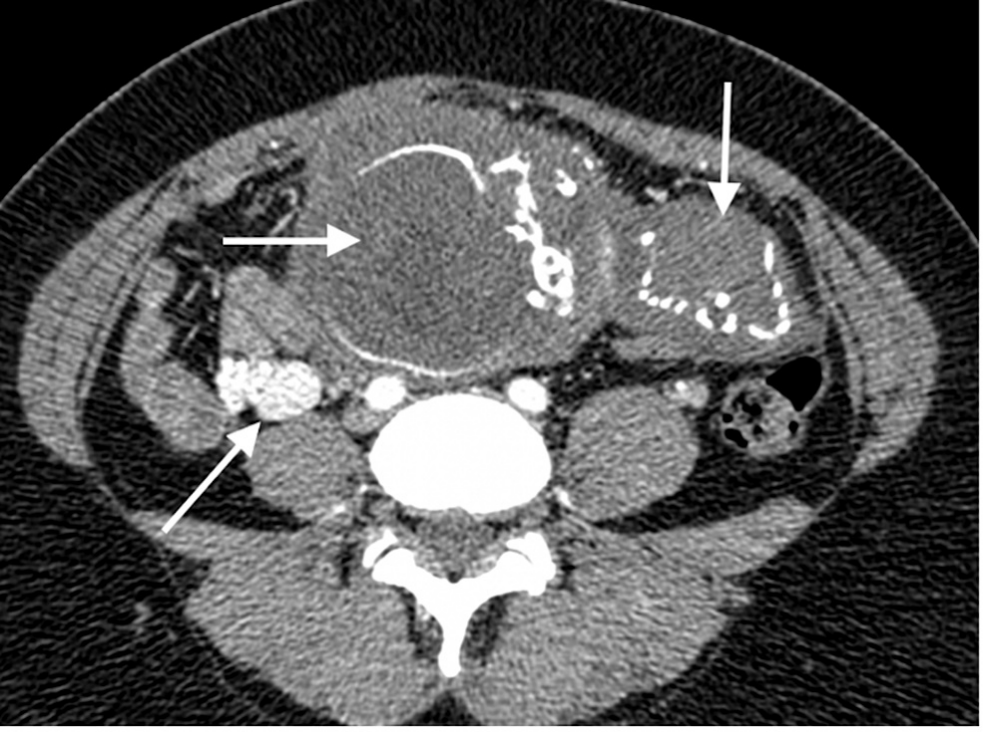
Axial, post-contrast CT, arterial phase demonstrating fetal head with hydrocephalus (horizontal arrow), fetal thoracic cavity located within the left side of the maternal abdomen (vertical arrow), and early filling of the engorged right ovarian vein (oblique arrow).

**Figure 2:**
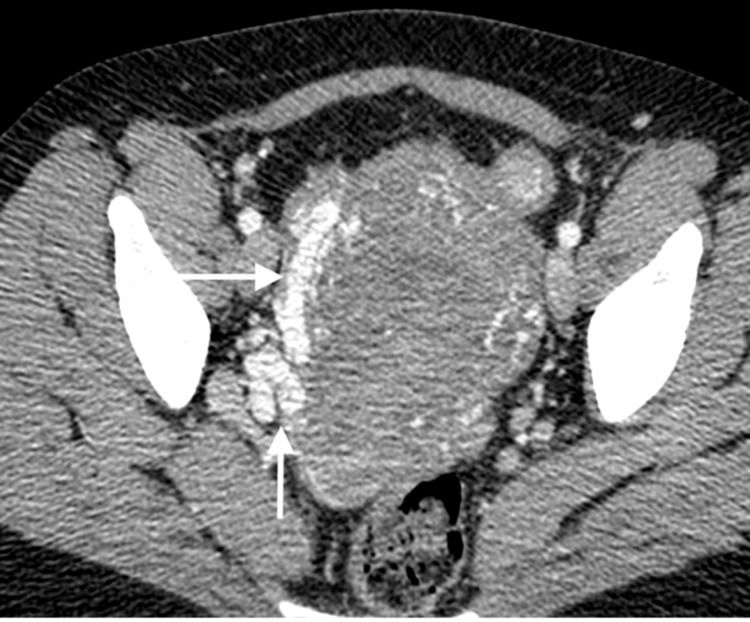
Axial, arterial phase post-contrast CT, through the pelvis, showing engorged, tortuous, right uterine artery (vertical arrow) and early filling of the right ovarian vein (horizontal arrow).

**Figure 3:**
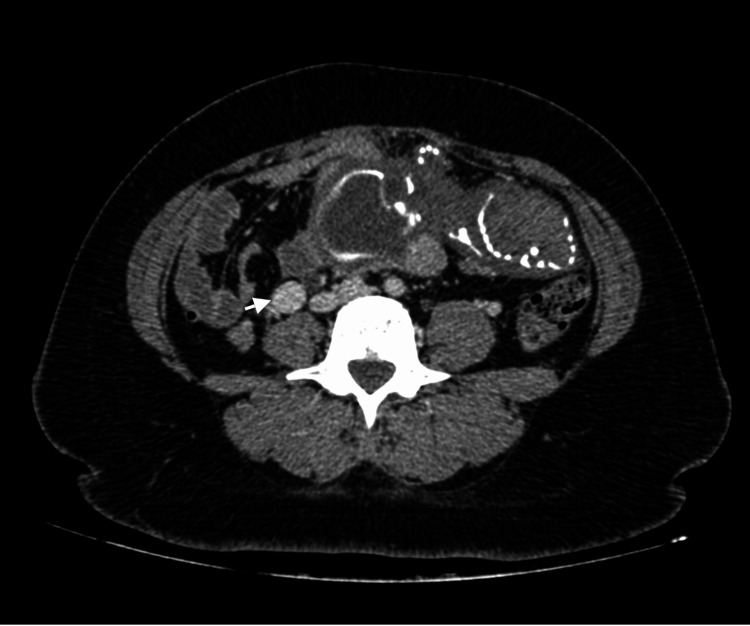
Axial, venous phase post-contrast CT, through the pelvis, showing engorged, tortuous right ovarian vein (arrow).

**Figure 4:**
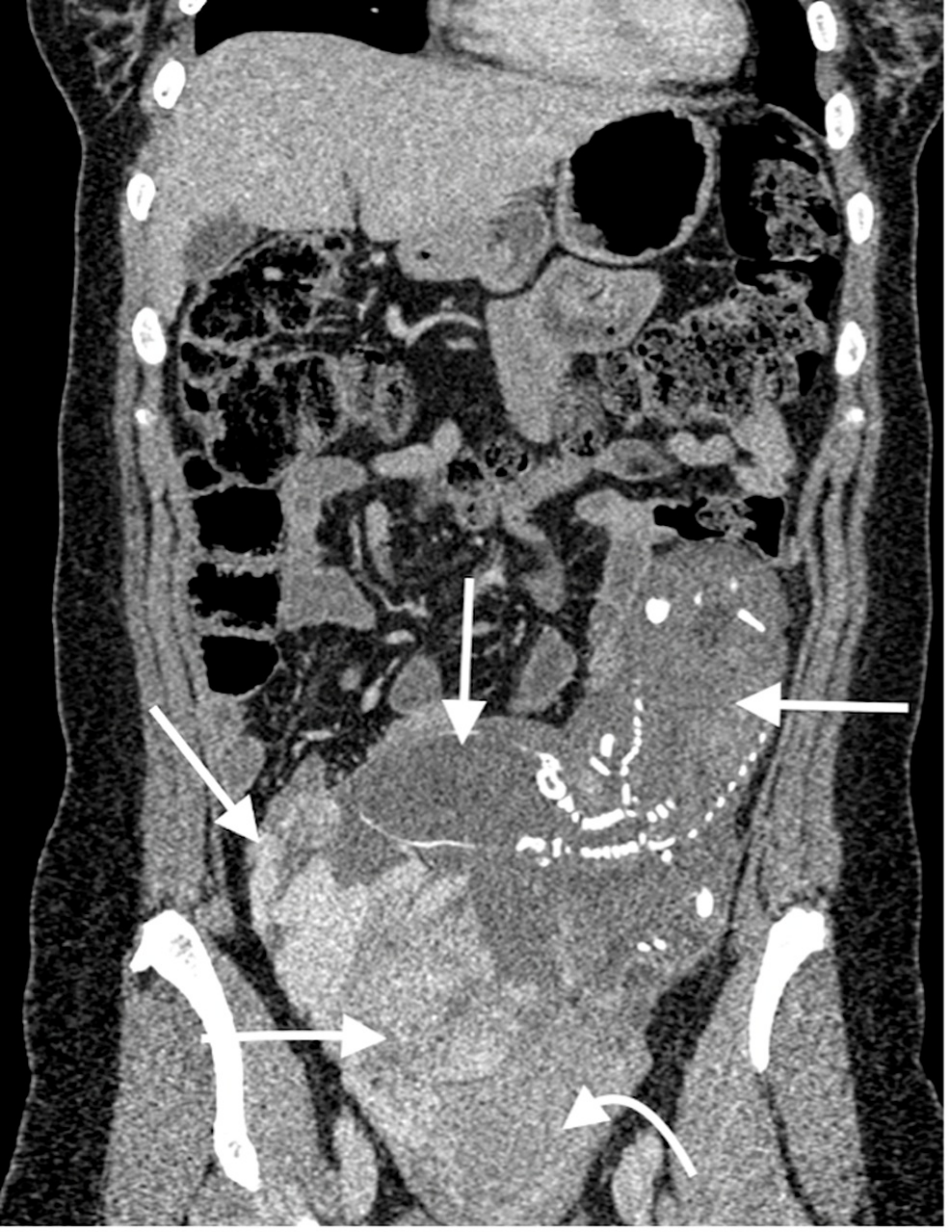
Coronal, post-contrast CT image showing scaphocephaly and hydrocephalus of the fetal head (vertical arrow), the fetal body located on the left side of peritoneal cavity (horizontal arrow pointing to right), uterine body (curved arrow), hypervascular placental tissue (horizontal arrow pointing to left), and early filling of the right ovarian vein (oblique arrow).

**Figure 5:**
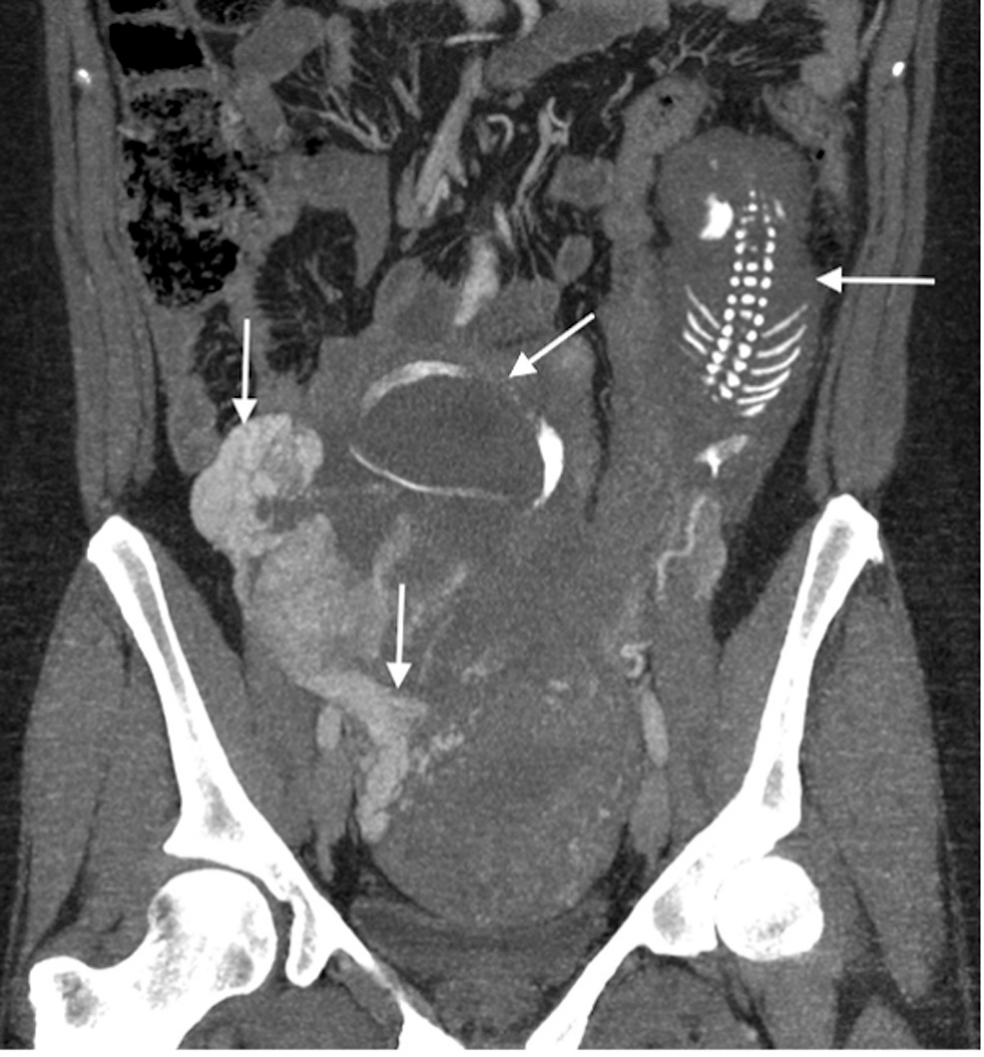
Coronal post IV contrast MIP image showing fetal head with scaphocephaly (oblique arrow), fetal body within the left paracolic gutter (horizontal arrow), and early filling of the right ovarian vein (vertical arrow). MIP: maximum intensity projection

Imaging confirmed an extrauterine pregnancy with the aforementioned abnormalities of the fetal head (Figures 1, 3, 4). There was extrauterine placental tissue closely related to the fetal vault, skull base, and neck as well as the uterine fundus and adjacent bowel (Figure 3). Incidentally, there was a large arteriovenous malformation in the right side, with arterial supply from branches of the right uterine artery and early opacification of the engorged right ovarian vein (Figures 1-5).

**Figure 6:**
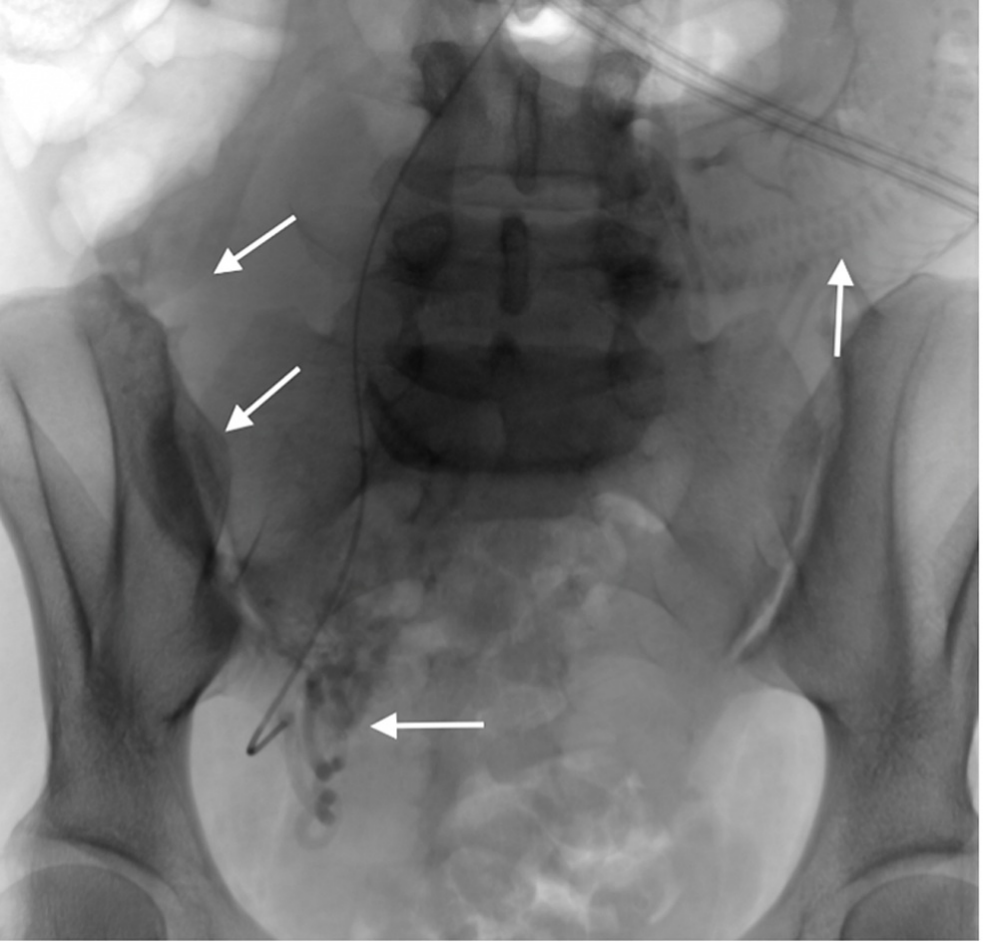
Right uterine artery angiogram showing the prominent tortuous right uterine artery (horizontal arrow) and early filling of the right ovarian vein (oblique arrows).

**Figure 7:**
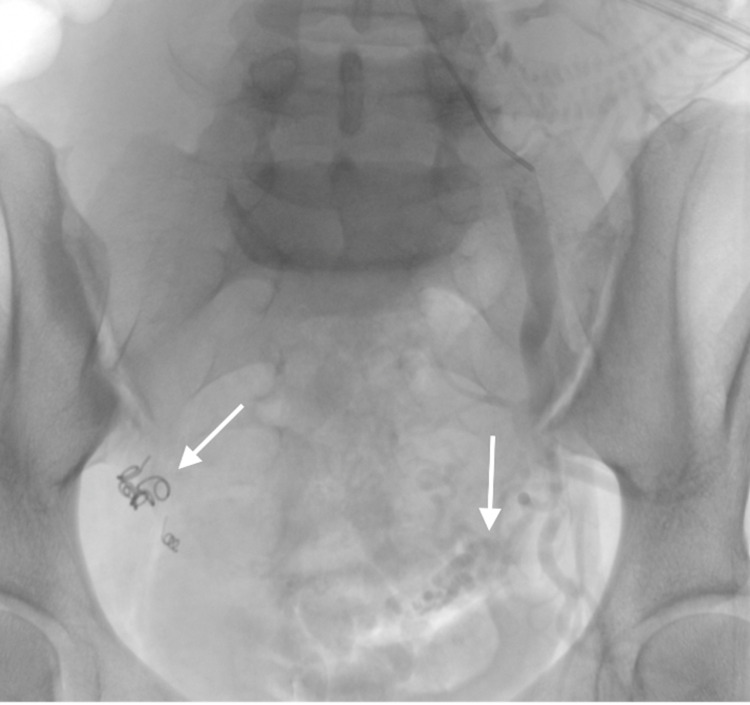
Left common iliac artery angiogram showing normal caliber left uterine artery (vertical arrow) and endovascular coils within the right uterine artery (oblique arrow).

Prior to surgical intervention, the patient underwent bilateral uterine artery coil embolization following which open laparotomy was performed (Figures 6, 7). The extrauterine fetus was confirmed, with head in the midline abutting the placenta and the body of fetus lying in the left paracolic gutter. The umbilical cord was ligated and the placenta was left in situ. The patient was subsequently placed on methotrexate, with satisfactory follow-up ultrasounds, and an unremarkable post-surgical clinical course.

## Discussion

An advanced abdominal pregnancy is defined as a pregnancy over 20 weeks gestation with fetus living or showing evidence of having once lived and developed, in the mother’s abdominal cavity, it is a rare form of ectopic pregnancy with an incidence ranging from one in 400 to one in 50,000 deliveries [1]. Abdominal pregnancies account for 1.4% of ectopic pregnancies [2,5].

The diagnosis of abdominal pregnancy requires confirmatory ultrasound imaging. However, approximately 50% of early abdominal pregnancies are missed on ultrasound, and as a result, MRI and CT provide excellent diagnostic information on abdominal pregnancies [6]. Additionally, the use of further cross-sectional imaging in hemodynamically stable patients can provide the advantages of giving clearer anatomic detail of the fetus, size and morphology of the placenta, its vascularization, and the relationship of the placenta to the adjacent intra-abdominal organs. The latter can allow preoperative planning, in order to decrease the morbidity and mortality associated with abdominal pregnancy [5].

CT imaging has an important role in the diagnosis and evaluation of extrauterine pregnancy but is limited by its limited role in assessment of fetal anatomy when compared to MRI. In our case, due to the advanced period of gestation, the presence of sonographically confirmed cranial abnormalities (scaphocephaly and hydrocephalus) and the unavailability of MRI, multidetector CT with IV contrast was used for further radiological assessment [7]. CT also played an important role in delineating the placental vasculature and evaluating the placental arteriovenous malformation, thus providing important information for interventional radiology planning. Furthermore, our case highlights the use of CT in the assessment of ovarian vein anatomy and engorgement. Dilated ovarian veins are quite common in the setting of pregnancy, as a result of increased blood flow to the ovarian veins [8]. It is typically seen in the late second and third trimesters [9].

MRI however, is the modality of choice, where available, and is particularly desirable where the utilization of ionizing radiation or iodinated contrast is inadvisable or not possible or desirable. It provides better anatomic detail as there is reduced interference by skeletal, fat and gas-filled maternal structure thus giving better fetal detail [10].

## Conclusions

The use of CT imaging can not only be diagnostic for abdominal pregnancies but can provide invaluable information on fetal anatomy and placental morphology/vascularity, especially when MRI is contraindicated or not available. Contrast-enhanced multidetector CT (CE-MDCT) can be used preoperatively to plan surgical and interventional radiological procedures, which can have a significant impact in decreasing morbidity and mortality associated with abdominal pregnancy.
